# Retraction Note: LncRNA LINC00337 sponges mir-1285-3p to promote proliferation and metastasis of lung adenocarcinoma cells by upregulating YTHDF1

**DOI:** 10.1186/s12935-024-03431-0

**Published:** 2024-07-14

**Authors:** Ru-nan Zhang, Dong-mei Wu, Li-ping Wu, Guo-wei Gao

**Affiliations:** Department of Radiation Oncology, Xinxiang Central Hospital, No.56 Jinsui Road, Xinxiang, Henan 453000 People’s Republic of China

**Retraction Note to:** Cancer Cell International (2021) 21:550

10.1186/s12935-021-02253-8

The Editors-in-Chief have retracted this article. After publication, concerns were raised about overlaps in several images, namely,

- Fig. 2C: The vector panel appears similar to the MG63/+anti-miR panel in Fig. 7A in a previously-published article by different authors [1];
- Fig. 2C: Linc00337 panel appears similar to the MG63/+NC panel in Fig. 7A in a previously-published article by different authors [1];
- Fig. 3B: the vector panel appears similar to the sh-circ-0008003 panel in Fig. 2D in a previously-published article by different authors [2];
- In Fig. 7B, the LINC00337 panel appears similar to the LINC01705 OE panel in Fig. 3D in a previously-published article by different authors [3];
- In Fig. 7B, the control and miR-1285-3p panels appear similar to the miR-NC and miR-186 5p mimics panels in Fig. 7E in a previously-published article by different authors [3].

The authors did not respond to a request to clarify. Therefore, the Editors lost confidence in the integrity of the article. The authors did not reply to correspondence from the Editors about this retraction.
